# Current Management of Polycystic Ovary Syndrome: From Bench to Bedside

**DOI:** 10.1155/2018/7234543

**Published:** 2018-11-14

**Authors:** Antonio Simone Laganà, Salvatore Giovanni Vitale, Marco Noventa, Amerigo Vitagliano

**Affiliations:** ^1^Department of Obstetrics and Gynecology, “Filippo Del Ponte” Hospital, University of Insubria, Varese, Italy; ^2^Department of General Surgery and Medical Surgical Specialties, University of Catania, Catania, Italy; ^3^Department of Woman and Child Health, University of Padua, Padua, Italy

Polycystic ovary syndrome (PCOS) affects 6–10% of women in reproductive age and is characterized by hyperandrogenism, insulin resistance, and chronic anovulation [1]. It is a heterogeneous syndrome with not completely understood etiology that is related to a complex interaction between metabolic, endocrine, genetic, and environmental factors. Increasing evidence suggests that insulin resistance and secondary hyperinsulinemia play a key synergistic role with hyperandrogenism in the development and maintenance of metabolic alterations and anovulation or irregular cycles in both obese and lean patients with PCOS [2]. On that basis, current treatment strategies aim at reducing insulin resistance in patients with PCOS and, consequently, to reach a reduction of compensatory hyperinsulinemia, improving metabolic and ovulatory features [3–5]. Insulin-sensitizer drugs are the recommended first-line therapy according to recent guidelines [6] for women with PCOS and metabolic abnormalities [7–9] with the aim at improving fertility [10–13], although physical activity and lifestyle change should be considered the first steps in overweight and obese PCOS patients to achieve weight loss [14, 15].

In this scenario, we are honored to introduce this special issue, which contains five articles that may shed new light on the topic. In particular, three articles are focused on metabolic disturbances in PCOS women: the first one (“Free Testosterone Reflects Metabolic as well as Ovarian Disturbances in Subfertile Oligomenorrheic Women”) found that sex hormone-binding globulin and calculated free testosterone are associated with both ovarian ultrasound and metabolic parameters, such as the body mass index (BMI) and insulin resistance, suggesting a pivotal role for androgen excess in PCOS-related subfertility and ovulatory dysfunction; the second article (“Pericardial Fat Relates to Disturbances of Glucose Metabolism in Women with the Polycystic Ovary Syndrome, but Not in Healthy Control Subjects”) found that pericardial fat measured using 1H-magnetic resonance spectroscopy and imaging is positively related to atherogenic lipid profiles, BMI, waist circumference, and liver fat in women with PCOS, suggesting it as a potential noninvasive tool to predict metabolic prognosis in this population; the third article (“Low-Dose Spironolactone-Pioglitazone-Metformin Normalizes Circulating Fetuin-A Concentrations in Adolescent Girls with Polycystic Ovary Syndrome”) highlights that a low-dose combination of insulin sensitizers and an antiandrogen is able to normalize fetuin-A levels in adolescent girls with PCOS. Considering that high levels of fetuin-A have been associated with greater risks for type 2 diabetes and with features of metabolic syndrome, this treatment may significantly reduce metabolic consequences and prevent acute events.

Besides these three articles related to metabolic disturbances and their treatment, another paper (“The Place of In Vitro Maturation in PCO/PCOS”) depicted a clear and accurate summary of available evidence regarding the optimization of culture media, laboratory protocols, pregnancy rates, and neonatal outcomes following in vitro maturation (IVM) of human oocytes in PCOS women, which are known to have a variable incidence of infertility and worse outcomes following assisted reproductive technology.

Finally, the last paper (“Uterine Artery Doppler in Pregnancy: Women with PCOS Compared to Healthy Controls”) investigated differences in the uterine artery pulsatility index (UtAPI) between pregnant women with PCOS and healthy controls and explored the possible effects of metformin on this parameter. Interestingly, the authors found that there was no difference in the UtAPI between women with PCOS and healthy controls in the first and second trimesters of pregnancy; in addition, metformin was not found to have an immediate effect on the UtAPI.

Overall, the manuscripts published in this special issue add significant and novel elements for the understanding of the etiology, pathophysiology, diagnosis, and treatment of this complex and multifaceted syndrome. We offer these new insights to the readers, hoping that they will stimulate further debate and address new fields of investigation in the next future.
